# Association between Dexmedetomidine Use and Mortality in Patients with COVID-19 Receiving Invasive Mechanical Ventilation: A U.S. National COVID Cohort Collaborative (N3C) Study

**DOI:** 10.3390/jcm13123429

**Published:** 2024-06-12

**Authors:** John L. Hamilton, Rachel Baccile, Thomas J. Best, Pankaja Desai, Alan Landay, Juan C. Rojas, Markus A. Wimmer, Robert A. Balk, on behalf of the N3C Consortium

**Affiliations:** 1 Rush Medical College, Rush University Medical Center, Chicago, IL 60612, USA; pankaja_desai@rush.edu (P.D.); alan_landay@rush.edu (A.L.); juan_rojas@rush.edu (J.C.R.); markus_a_wimmer@rush.edu (M.A.W.); 2 Center for Health and the Social Sciences, University of Chicago, Chicago, IL 60637, USA; rbaccile@bsd.uchicago.edu (R.B.); tbest3@bsd.uchicago.edu (T.J.B.)

**Keywords:** coronavirus disease 2019 (COVID-19), dexmedetomidine, invasive mechanical ventilation, mortality, severe acute respiratory syndrome coronavirus 2 (SARS-CoV-2)

## Abstract

(1) **Background/Objectives**: Dexmedetomidine is a sedative for patients receiving invasive mechanical ventilation (IMV) that previous single-site studies have found to be associated with improved survival in patients with COVID-19. The reported clinical benefits include dampened inflammatory response, reduced respiratory depression, reduced agitation and delirium, improved preservation of responsiveness and arousability, and improved hypoxic pulmonary vasoconstriction and ventilation-perfusion ratio. Whether improved mortality is evident in large, multi-site COVID-19 data is understudied. (2) **Methods**: The association between dexmedetomidine use and mortality in patients with COVID-19 receiving IMV was assessed. This retrospective multi-center cohort study utilized patient data in the United States from health systems participating in the National COVID Cohort Collaborative (N3C) from 1 January 2020 to 3 November 2022. The primary outcome was 28-day mortality rate from the initiation of IMV. Propensity score matching adjusted for differences between the group with and without dexmedetomidine use. Adjusted hazard ratios (aHRs) for 28-day mortality were calculated using multivariable Cox proportional hazards models with dexmedetomidine use as a time-varying covariate. (3) **Results**: Among the 16,357,749 patients screened, 3806 patients across 17 health systems met the study criteria. Mortality was lower with dexmedetomidine use (aHR, 0.81; 95% CI, 0.73–0.90; *p* < 0.001). On subgroup analysis, mortality was lower with earlier dexmedetomidine use—initiated within the median of 3.5 days from the start of IMV—(aHR, 0.67; 95% CI, 0.60–0.76; *p* < 0.001) as well as use prior to standard, widespread use of dexamethasone for patients on respiratory support (prior to 30 July 2020) (aHR, 0.54; 95% CI, 0.42–0.69; *p* < 0.001). In a secondary model that was restricted to 576 patients across six health system sites with available PaO_2_/FiO_2_ data, mortality was not lower with dexmedetomidine use (aHR 0.95, 95% CI, 0.72–1.25; *p* = 0.73); however, on subgroup analysis, mortality was lower with dexmedetomidine use initiated earlier than the median dexmedetomidine start time after IMV (aHR, 0.72; 95% CI, 0.53–0.98; *p* = 0.04) and use prior to 30 July 2020 (aHR, 0.22; 95% CI, 0.06–0.78; *p* = 0.02). (4) **Conclusions**: Dexmedetomidine use was associated with reduced mortality in patients with COVID-19 receiving IMV, particularly when initiated earlier, rather than later, during the course of IMV as well as use prior to the standard, widespread usage of dexamethasone during respiratory support. These particular findings might suggest that the associated mortality benefit with dexmedetomidine use is tied to immunomodulation. However, further research including a large randomized controlled trial is warranted to evaluate the potential mortality benefit of DEX use in COVID-19 and evaluate the physiologic changes influenced by DEX that may enhance survival.

## 1. Introduction

Severe acute respiratory syndrome coronavirus 2 (SARS-CoV-2) has resulted in over 7 million deaths worldwide as of March 2024 [1]. Mortality in critically ill patients with coronavirus disease 2019 (COVID-19) is high [2,3,4,5], so there is a need to improve survival in critically ill patients with COVID-19.

Dexmedetomidine (DEX) is an alpha-2 adrenergic receptor (α2-AR) agonist that was introduced in 1999 as a sedative in the intensive care unit (ICU) for patients receiving mechanical ventilation [6]. Since introduction, interest has mounted over whether DEX improves outcomes including survival compared to gamma-aminobutyric acid (GABA) receptor ligand sedatives such as propofol or benzodiazepines for ICU patients [6,7]. One of the foundational reasons for this interest is the immunomodulatory effects of DEX [6,8,9]. While there is a rationale for targeting the inflammatory response as a survival strategy for ICU patients with sepsis or acute respiratory distress syndrome (ARDS), randomized controlled trials (RCTs) have demonstrated mixed outcomes with anti-inflammatory pharmacologic strategies such as corticosteroids [10] or DEX [7] outside of the COVID-19 population. The COVID-19 pandemic created a global urgency for investigations, leading to improved mortality outcomes for patients with SARS-CoV-2 infection. A key finding was that COVID-19 deaths are in part attributed to an inflammatory response [11], and RCTs demonstrated a reduction in mortality with corticosteroid use among those receiving IMV or oxygen support [12,13].

No large RCTs have evaluated DEX use in COVID-19 outcomes. The potential for DEX to improve COVID-19 outcomes has been proposed through dampening the inflammatory response [14,15,16] such as through sympatholytic and vagomimetic pathways [6,14,15,16], maintaining endothelial cell junction and microcirculatory integrity [17,18,19] as well as other direct and indirect effects on immune cells and other cells types [14,15,16,20]. Other reported benefits of DEX as a sedative option include the lack of significant respiratory depression [21], analgesic properties with an opioid-sparing effect [22], reduced agitation and delirium [23], preserving a degree of responsiveness and arousability [24] as well as potential improvements in hypoxic pulmonary vasoconstriction and the ventilation–perfusion ratio [15,25].

DEX is reported to improve COVID-19 outcomes including improved oxygen saturation (SpO_2_) [25], improved partial pressure of arterial oxygen to the fraction of inspired oxygen (PaO_2_/FiO_2_) [26], shortened duration of mechanical ventilation [27], and reduced mortality [28,29]. However, these investigations have been limited in both scope and size. For this study, we utilized the National Institutes of Health’s National COVID Cohort Collaborative (N3C) Data Enclave that houses electronic health record (EHR) data for over 6 million COVID-19 positive patients across over 70 health systems in the United States [30,31]. In this retrospective multi-center cohort study, we hypothesized that DEX use would be associated with improved survival in critically ill patients with COVID-19 receiving IMV in the N3C database.

## 2. Materials and Methods

### 2.1. Study Design

**Figure 1:**
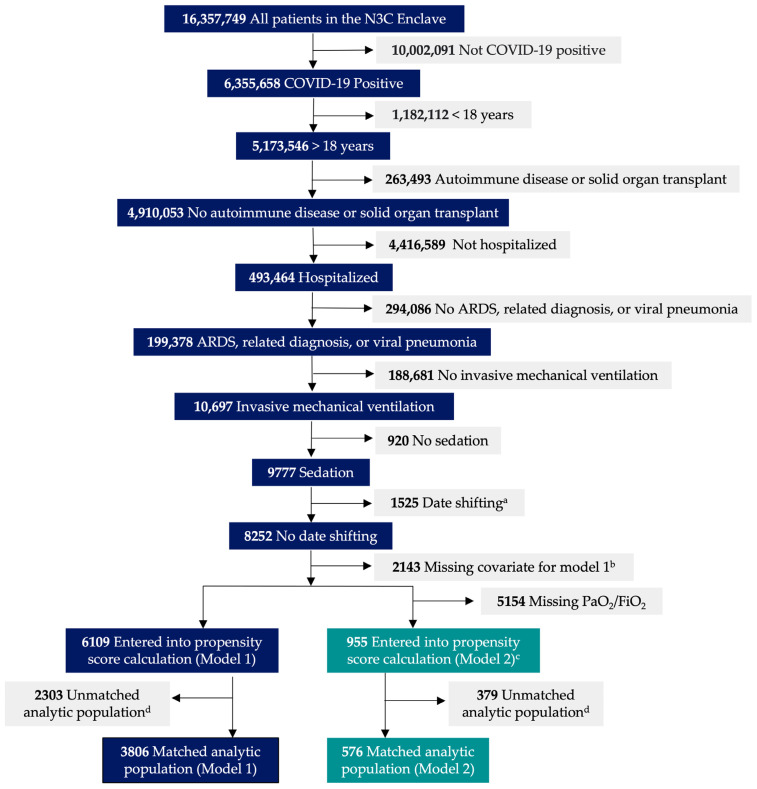
Flow diagram depicting the phases of enrollment, exclusion, and data analysis for Model 1 and Model 2. ^a^ Patients with implausible data points such as a COVID-19 diagnosis in 2017 or individuals in whom the date of death predated their date of hospital admission were excluded. ^b^ If a patient was missing a specific covariate for the Cox model, they were excluded for complete case analysis. These specific covariates included age, body mass index, modified Charlson comorbidity index, and modified Sequential Organ Failure Assessment score (central nervous system and respiratory component removed). ^c^ Compared to Model 1, additional Model 2 covariates included PaO_2_/FiO_2_ and modified Sequential Organ Failure Assessment score with respiratory component added for complete cases analyses. ^d^ Unmatched patients between the dexmedetomidine group and no dexmedetomidine group on propensity score calculation were removed.

This retrospective multi-center cohort study was performed using EHR data in the N3C Data Enclave hosted by the National Center for Advancing Translational Sciences (NCATS). Patients were included if they had: (i) a diagnosis of COVID-19 or laboratory confirmed SARS-CoV-2 on polymerase chain reaction or antigen test results [32]; (ii) hospitalization within 21 days of first positive COVID-19 indication; (iii) ARDS, related diagnosis, or viral pneumonia; and (iv) received IMV and sedation. The median time between first COVID-19 indication and hospitalization are provided in Table S1. Exclusion criteria included: (i) younger than 18 years of age; (ii) diagnosis of autoimmune disease; and (iii) history of solid organ transplant. Phases of enrollment, exclusion, and data analysis are provided in Figure 1. This study was approved by the institutional review boards at Rush University Medical Center (22032001-IRB01), University of Chicago (IRB22-0681), and the NCATS N3C Data Access Committee (DUR-DODE010). The authors used the Strengthening the Reporting of Observational Research Studies in Epidemiology (STROBE) guidelines [33].

### 2.2. Data Collection

The N3C aggregates and harmonizes EHR data for patients with laboratory confirmed or suspected COVID-19 during any encounter after 1 January 2020 from data partners (i.e., sites) across the U.S. [30,31]. Within the N3C, sites are asked to upload two years of health histories before the earliest COVID-19 test date for each patient. The design, sampling, and harmonization methods used in the N3C Enclave have been described previously [31]. This investigation used the N3C Limited Dataset (LDS) [30], which retained the dates of clinical services without shifting for all patients meeting the inclusion criteria. External (i.e., non-site) mortality data were incorporated by the N3C Privacy-Preserving Record Linkage [34]. Data collection within the N3C Data Enclave was performed from 1 January 2020 to 3 November 2022.

### 2.3. Outcomes

Cohorts included patients that received DEX (DEX group) and patients that did not receive DEX (no DEX group). The DEX group was defined as patients that received DEX between the start time of IMV and 28 days thereafter. Patients that started DEX before IMV were included but only if DEX was continued between the start of IMV and 28 days later. The primary outcome was 28-day mortality rate from the initiation of IMV. This outcome was calculated by performing propensity score matching between the DEX and no DEX group followed by multivariable Cox regression analysis. Based on our previous investigation [28], we hypothesized that DEX use would be associated with a lower 28-day mortality rate.

### 2.4. Covariates

Propensity score matching between the DEX and no DEX group was performed to adjust for significant covariate differences at hospital admission and ICU variables; pre-propensity score matched covariates at hospital admission and in the ICU are provided in Tables S2 and S3. A significant difference between groups was identified as a standardized mean difference (SMD) > 0.2 [35]. In addition, relevant clinical variables were identified a priori that could have the greatest confounding influence on mortality and were included in the multivariable Cox (proportional hazards) regression analysis for 28-day mortality, in addition to DEX use, as described previously [28]. These variables included factors at hospital admission: age, body mass index (BMI), and modified Charlson comorbidity index (mCCI). The mCCI was calculated as described by Quan et al. (2011) [36]. Additional variables included PaO_2_/FiO_2_ and modified Sequential Organ Failure Assessment (mSOFA) scores at the start time of IMV as well as dexamethasone and remdesivir use during hospitalization [28]. The PaO_2_/FiO_2_ ratio and mSOFA score were calculated as the worst value over 24 h from the start time of IMV [37]. In all mSOFA score calculations, the central nervous system component was removed, since patients were assessed while under sedation. Within the Cox models, DEX use was treated as a time-varying covariate to adjust for immortal time bias [28,38].

Interleukin-6 (IL-6) receptor antagonists (tocilizumab and sarilumab), Janus kinase (JAK) inhibitors (baricitinib and tofacitinib), and COVID-19 vaccination status prior to a COVID-19 diagnosis were initially identified as covariates due to their immunomodulatory effects and potential influences on mortality. However, between 0 and 6% of the patients received these treatments in either group in our models in the N3C database with no significant differences between groups (Tables S4–S6) and were removed as covariates and used in propensity score matching and Cox regression. Following propensity score matching, these variables remained between 0 and 6% between groups with no significant difference between groups (Tables S7–S9).

### 2.5. Subgroup Analysis

As a subgroup analysis, evaluation of the influence of the earlier or later initiation of DEX on survival was performed as previously described [28]. Earlier DEX start time was defined as DEX use initiated earlier than the median start time of DEX use relative to the start of IMV amongst the entire DEX group; later start time was defined as DEX use after the median start time. We hypothesized that earlier DEX start times relative to IMV initiation would be associated with a greater reduced risk of death, as previously found [28], which may be due to promoting early immunomodulatory benefits around the time of IMV, dampening COVID-19 progression.

This investigation was performed between 1 January 2020 and 3 November 2022. Starting in July 2020, there was a shift toward the standard usage of corticosteroids, in particular dexamethasone, at the time of IMV or oxygen support in patients with COVID-19, based on the RCT findings [12,13]. Since we hypothesized that DEX may have immunomodulatory benefits in critically ill patients with COVID-19, we questioned whether the shift in treatment protocols, starting around July 2020 incorporating dexamethasone, a powerful immunosuppressant, around the time of IMV, influenced the associated mortality benefit of DEX; dexamethasone may provide powerful immunosuppression to a degree where the addition of the immunomodulatory benefits of DEX are diminished. To further evaluate the impact of the current dexamethasone guidelines on the association between DEX use and mortality, we used the same propensity score matching and Cox regression models incorporating patients either prior to 30 July 2020 (pre-dexamethasone era) or post 30 July 2020 (current dexamethasone era).

### 2.6. Statistical Analysis

Data acquisition and analysis were performed using Palantir Foundry (Palantir Technologies Inc., Denver, CO, USA), Python (Python Software Foundation), version 3.6, and R, version 4.0.2 (R Foundation for Statistical Computing) using the survival package [39,40] hosted within the N3C Enclave. Continuous variables are presented as the mean and standard deviation (SD). Categorical variables were calculated as the number and percentage of patients; if the number was less than 20, the number and percentage was not reported for reasons of person privacy and N3C policy. For all variables, an SMD was calculated.

The 28-day mortality rate as an outcome was evaluated using propensity score matching followed by Cox regression. Propensity score matching was performed using 1:1 nearest neighbor matching, without replacement, with a caliper [41]. Model 1 (primary model) adjusted for variables at hospital admission and ICU variables with an SMD > 0.2 with propensity score matching and further adjusted a priori selected covariates in Cox regression including: (1) DEX use as a time-varying covariate, (2) age, (3) BMI, (4) mCCI, (5) mSOFA (central nervous system and respiratory component removed), (6) dexamethasone use, and (7) remdesivir use. The Model 2 (secondary model) covariates were identical to Model 1 with the addition of PaO_2_/FiO_2_ and the mSOFA respiratory component in the Cox regression model. The majority of sites in the N3C Enclave did not have PaO_2_/FiO_2_ data available, primarily because the N3C did not require that sites submit data on ventilator settings, where FiO_2_ is often documented. As of fall 2022, only six sites nationwide in the N3C Data Enclave had PaO_2_/FiO_2_ data, with 42.9% of that data coming from a single site. Due to this limitation, we removed the requirement of PaO_2_/FiO_2_ data for our primary model (Model 1). In both models, adjustment for site differences was performed by including a categorical variable with levels for each data partner that represented > 5% of our population and another level for all other sites grouped together [42].

Simple imputation using the mean of the immediate preceding and succeeding most severe value over 24 h was used for missing values for the component mSOFA scores (Model 1 and 2) and PaO_2_/FiO_2_ (Model 2) within the 24 h time period of interest—from initiation of IMV [37]. Within both models, a complete case analysis was performed, with exclusion of any patient with a missing covariate or implausible data points such as a COVID-19 diagnosis in 2017 or individuals in whom the date of death predated their date of hospital admission within the N3C database (Figure 1). Mortality outcome data over 28 days are presented as adjusted hazard ratios (aHRs) and 95% confidence intervals (CIs) with *p* < 0.05 considered significant.

## 3. Results

For the primary analysis (Model 1), among the 16,357,749 patients screened, there were a total of 17 health systems that included 6109 patients, with 3002 patients in the DEX group and 3107 patients in the no DEX group meeting the inclusion and exclusion criteria. Following propensity score matching, there were 1903 patients in the DEX group and 1903 patients in the no DEX group (Figure 1). The mean age in Model 1 was 62.6 years; 61.6% were male, 17.4% were Black, 59.3% were White, and 17.7% were Hispanic. In the restricted analysis (Model 2), where only sites that provided PaO_2_/FiO_2_ data were included, there were six health system sites that included 955 patients (Figure 1), with 615 patients in the DEX group and 340 patients in the no DEX group meeting the inclusion and exclusion criteria. Following propensity score matching, there were 288 patients in both the DEX and no DEX group (Figure 1). The mean age in Model 2 was 63.6 years; 63.5% were male, 4.0% were Black, 77.8% were White, and 12.8% were Hispanic. 

### 3.1. Covariate Balance before and after Propensity Score Matching

In Model 1, the SMDs before propensity score matching were considered significant (>0.2) in one out of twenty-eight variables at hospital admission (3.6%) and five out of twenty-five ICU variables (20.0%) (Tables S2 and S3). In Model 2, the SMDs before propensity score matching were considered significant (>0.2) in two out of twenty-eight variables at hospital admission (7.1%) and six out of twenty-six ICU variables (23.1%) (Tables S2 and S3). After propensity score matching was performed, all variables at hospital admission and the ICU variables were similar between groups, with an SMD less than 0.2 (Table 1 and Table 2).

### 3.2. Outcomes

Of the 3806 patients included in Model 1 following propensity score matching, 57.2% died between the start of IMV and 28 days thereafter. The percentage of patients that died was lower in the DEX group compared to the no DEX group (47.4% vs. 67.0%; relative risk reduction, 29.3%, *p* < 0.001). In Model 1, the 28-day mortality rate was lower with DEX use on multivariable regression (aHR, 0.81; 95% CI, 0.73–0.90; *p* < 0.001). Similarly, the 28-day mortality rate was lower with DEX use on univariable regression (aHR, 0.90; 95% CI, 0.81–0.99; *p* = 0.04) (Table 3). Of the 576 patients included in Model 2 following propensity score matching, 55.4% died between the start of IMV and 28 days thereafter. The percentage of patients that died was lower in the DEX group compared to the no DEX group (49.7% vs. 61.1%; relative risk reduction, 18.7%, *p* < 0.01), but DEX use was not associated with a significantly lower 28-day mortality rate with multivariable regression (aHR, 0.95; 95% CI, 0.72–1.25; *p* = 0.73) or univariable regression (aHR, 1.05; 95% CI, 0.80–1.36; *p* = 0.74 (Table 3). The survival curves between cohorts in Model 1 and Model 2 using multivariable regression are provided in Figures S1 and S2. The aHRs for all covariates in Model 1 and Model 2 are provided in Tables S10 and S11.

### 3.3. Subgroup Analysis

**Figure 2:**
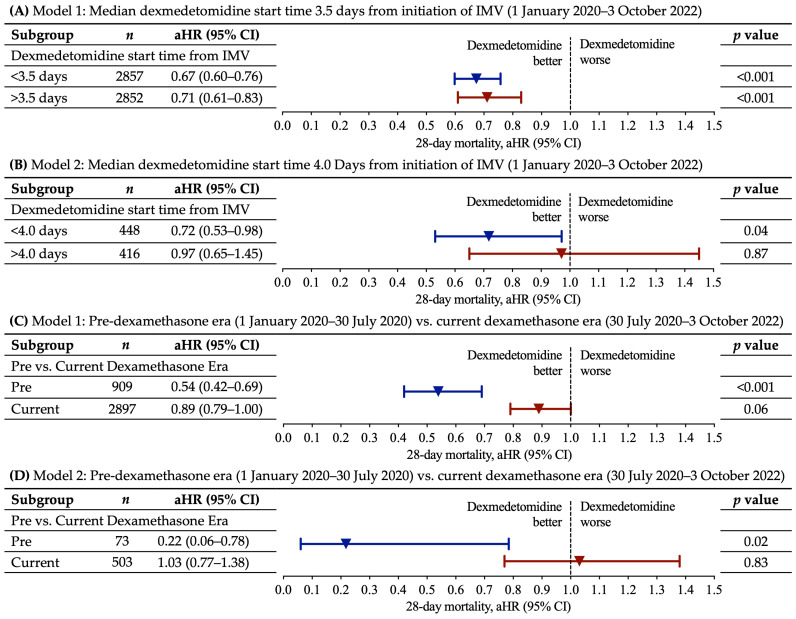
Subgroup analysis examining 28-day mortality rate based on start time of dexmedetomidine as well as dexmedetomidine use pre-dexamethasone era vs. current dexamethasone era. (**A**,**B**) Shown are the prespecified subgroup analyses for Model 1 and Model 2 by dexmedetomidine start times pre or post median start time of dexmedetomidine from the start of invasive mechanical ventilation (IMV). The median start time of DEX is shown for both Model 1 and Model 2. (**C**,**D**) Shown are the prespecified subgroup analyses for Model 1 and Model 2 by dexmedetomidine use pre-dexamethasone era (1 January 2020 to 30 July 2020) vs. dexmedetomidine use during the current dexamethasone era (30 July 2020 to 3 November 2022). Within the models, propensity score matching and multivariable Cox regressions were performed, and the total number of patients are displayed for each subgroup. The adjusted hazard ratios for each subgroup are plotted as an inverted triangle, and 95% CIs are plotted as horizontal lines. *p* values < 0.05 are considered significant and correspond to an aHR and 95% CI below 1. Abbreviations: aHR = adjusted hazard ratio; 95% CI = 95% confidence interval.

The median start time of DEX from the initiation of IMV was 3.5 days (interquartile range (IQR), 7.0 days) in Model 1 and 4.0 days (IQR, 5.0 days) in Model 2. Within the respective models, we evaluated whether the 28-day mortality rates were influenced by DEX start times earlier or later than the median start time of DEX from the initiation of IMV. Earlier DEX start time was associated with a reduction in the 28-day mortality rate in both Model 1 (aHR, 0.67; 95% CI, 0.60–0.76; *p* < 0.001) and Model 2 (aHR, 0.72; 95% CI, 0.53–0.98; *p* = 0.04) (Figure 2A,B). Later DEX start time was associated with a lower mortality rate in Model 1 (aHR, 0.71; 95% CI, 0.61–0.83; *p* < 0.001) but not Model 2 (aHR, 0.97; 95% CI, 0.65–1.45; *p* = 0.87) (Figure 2A,B). In both models, earlier DEX start times compared to later DEX start times were associated with a lower aHR (Figure 2A,B). The aHRs for all covariates in this subgroup analyses are provided in Tables S10 and S11. The unadjusted percentage of patients who died in the DEX group in Model 1 was 56.7% (early treatment) and 38.0% (late treatment) compared to 67.0% in the no DEX group (Table S12); in Model 2, the unadjusted percentage of patients who died in the DEX group was 54.4% (early treatment) and 43.8% (late treatment) compared to 61.1% deaths in the no DEX group (Table S12).

To further evaluate the impact of the current dexamethasone guidelines on the association between DEX use and mortality, we used the same Model 1 and Model 2 incorporating patients either pre-dexamethasone era (1 January 2020 to 30 July 2020) or during the current dexamethasone era (30 July 2020 to 3 November 2022). There were substantially lower aHRs for DEX use in the pre-dexamethasone era as opposed to the current dexamethasone era in both Model 1 (pre-dexamethasone era aHR 0.54; 95% CI, 0.42–0.69; *p* < 0.001 vs. current dexamethasone era aHR 0.89; 95% CI, 0.79–1.00; *p* = 0.06) and Model 2 (pre-dexamethasone era aHR 0.22; 95% CI 0.06–0.78; *p* = 0.02 vs. current dexamethasone era 1.03; 95% CI, 0.77–1.38; *p* = 0.83) (Figure 2C,D). The aHRs for all covariates in this subgroup analyses are provided in Tables S13–S16. The unadjusted percentage of patients who died in the DEX group vs. no DEX group in Model 1 was as follows: 36.2% DEX group vs. 62.7% no DEX group (pre-dexamethasone era) and 51.2% DEX group vs. 68.2% no DEX group (current dexamethasone era) (Table S17). In Model 2, the percentage of patients who died in the current dexamethasone era was 51.0% DEX group and 61.4% no DEX group; in the pre-dexamethasone era, the no DEX group had 59.5% deaths, and the DEX group had less than 20 patients that died, and therefore the percentage was masked to protect patient privacy as per the N3C policy (Table S17).

## 4. Discussion

In this retrospective multi-center cohort study including 3806 patients (Model 1; primary model) in the N3C database with COVID-19 receiving IMV, DEX use was associated with a lower aHR for 28-day mortality from the start time of IMV. Within Model 1, on subgroup analysis, earlier initiation of DEX—DEX use within the median of 3.5 days from the start of IMV—was associated with a lower aHR; furthermore, DEX use prior to 30 July 2020 was associated with a lower aHR. In the secondary model (Model 2) that included 576 patients, with restriction to clinical sites providing PaO_2_/FiO_2_ data, mortality was not lower with DEX use; however, similar to Model 1, on subgroup analysis, associated mortality was lower with earlier initiation of DEX use and DEX use prior to 30 July 2020.

The differences between Model 1 and Model 2 with regard to overall associated mortality outcomes between the DEX and no DEX group could be multifactorial. Model 2 was restricted to substantially fewer patients and clinical sites due to the requirement of PaO_2_/FiO_2_ data, which was limited across many sites in the N3C database. Furthermore, these PaO_2_/FiO_2_ data as well as further incorporation of these data by the addition of the respiratory component in the mSOFA score were factored into the propensity score matching and multivariable Cox regression in Model 2. Therefore, while Model 2 had fewer patients and sites, it better accounted for COVID-19 severity. Intriguingly, while both Model 1 and Model 2 showed reduced mortality with early DEX use or use prior to 30 July 2020, the dichotomy in mortality outcomes compared to later DEX initiation time or DEX use post 30 July 2020 was greater within Model 2 than Model 1. As previously discussed, this dichotomy may be related to differences in the sample size, total clinical sites, or key covariates incorporated into the model.

Our findings are consistent with other observational studies that found lower mortality with DEX use in critically ill patients with COVID-19 [28,29]. The lower 28-day mortality rate with earlier DEX use relative to IMV is consistent with our previous investigation at Rush University System for Health (RUSH) hospitals [28]. Lower mortality with earlier DEX use might be attributed to the immunomodulatory effects of DEX [6,14,15,16,17,18,19]. Failure of the initial clearance of SARS-CoV-2 infection and/or a dysfunctional immune response can result in an inflammatory cascade, contributing to severe lung pathology and a major systemic inflammatory response [43]. Earlier initiation of DEX use relative to start time of IMV may further limit organ dysfunction, irreversible organ damage, and death.

If DEX improves COVID-19 mortality outcomes through immunomodulatory effects, we hypothesized that the incorporation of immunosuppressant corticosteroids, in particular dexamethasone, around the time of respiratory support would reduce the association between DEX and lower mortality—dexamethasone may suppress the immune response to a degree where immunomodulation by DEX provides limited added benefit. Starting in July 2020, there was a shift toward the standard usage of corticosteroids, in particular dexamethasone, at the time of IMV or oxygen support in COVID-19, based on RCT findings [12,13]. In dramatic fashion, the associated mortality benefit with DEX was the greatest during the pre-dexamethasone era (prior to 30 July 2020) in both Model 1 and Model 2. These results potentially suggest that DEX and dexamethasone might have overlapping effects that reduce mortality such as through dampening the inflammatory response.

The use of α2-AR agonists such as DEX have been widely reported to dampen the inflammatory response [14,15,16] such as through sympatholytic and vagomimetic pathways [6,14,15,16] and other direct and indirect effects on immune cells and other cells types [14,15,16,20]. Furthermore, α2-AR agonists have direct effects on the vascular endothelium [17,18,19] with the limitation of circulating leukocyte (neutrophil) extravasation to the site of inflammatory stimulus and tissue [17,44]. The use of corticosteroids, while beneficial around the time of respiratory support in COVID-19, has been of concern as a “double-edged sword” due to the potential to reduce anti-viral immunity and viral clearance through immunosuppression [45,46,47]. For specific infectious diseases, due to concerns of reduced pathogen clearance, DEX may provide an immunomodulatory effect that is overall less immunosuppressive than corticosteroids; however, this was not addressed in this investigation.

While the lowest aHRs for mortality with earlier DEX use relative to IMV and use prior to the current dexamethasone era may suggest an immunomodulatory effect, we cannot rule out alternative potential benefits of DEX use influencing reduced mortality. Other reported benefits of DEX as a sedative option, which could influence mortality, include the following: lack of significant respiratory depression [21], reduced agitation and delirium [23], preserving a degree of responsiveness and arousability [24], and potential improvements in hypoxic pulmonary vasoconstriction and ventilation–perfusion ratio [15,25].

Other studies have predominately evaluated short-term outcomes of DEX use in patients with COVID-19 with severe or critical illness [25,26,27]. A case report found improvement in oxygen saturation (SpO2) on high flow nasal cannula with DEX use in the emergency room setting [25]. In the ICU, the initiation of DEX was associated with improvement in PaO_2_/FiO_2_ in a retrospective cohort analysis [26]. In a small double-blinded controlled clinical trial in the ICU, the DEX group compared to the propofol control group had a shorter duration of mechanical ventilation [27]. Clinical trials listed underway investigating DEX use in COVID-19 outcomes include NCT04358627, NCT04413864, and NCT05233605 (ClinicalTrials.Gov, accessed on 5 April 2024). Alternatively, the α2-AR agonist clonidine, which is used as an antihypertensive, appeared to limit the progression of moderate to severe COVID-19 in a case series, when initiated before or at the time of the requirement of oxygenation or hospitalization [48].

This study had a number of strengths including being a multi-center design, substantial sample size, with rigorous control for differences between the DEX and no DEX groups using propensity score matching and multivariable Cox regression. Following propensity score matching, there were no significant differences between the DEX and no DEX group with regard to variables at hospital admission or ICU variables. Furthermore, Cox regression further adjusted for a priori selected covariates that could have had the most confounding influence on mortality outcomes, which were the following: age, BMI, mCCI, mSOFA, dexamethasone use, remdesivir use as well as the addition PaO_2_/FiO_2_ and the incorporation of the respiratory component in the mSOFA score in Model 2. Furthermore, within the Cox regression models, DEX use was treated as a time-varying covariate to adjust for immortal time bias [28,38].

This study also had certain limitations. This work was retrospective in nature, and the N3C database has limitations with regard to the covariates that can be assessed. Due to ventilator setting data being available in the N3C database at only a limited number of sites, we removed the requirement of PaO_2_/FiO_2_ from the primary model (Model 1) but included PaO_2_/FiO_2_ in the restricted secondary model (Model 2); this restriction decreased the overall sample size and number sites in Model 2. Sample size was also further restricted on subgroup analysis in particular for Model 2 when assessing the pre-dexamethasone era vs. current dexamethasone era. The pre-dexamethasone era was the most restricted in sample size and took place from the initial start of the pandemic in the U.S. from 1 January 2020 to 30 July 2020, while the current dexamethasone era in this study (30 July 2020–3 October 2022) had more patients enrolled over a greater window of time. Prone positioning use was initially part of the variables selected for the Cox models as previously described [28] due to its reported benefits of improved oxygenation, which might improve survival in patients with COVID-19 [49]. However, these data were not available within the N3C Data Enclave. The ability to look at dose-dependent associated mortality outcomes with DEX was initially planned for assessment; however, this assessment was not possible in the N3C database. Alwakeel et al. (2024) was able to incorporate the association of individual sedative (propofol, ketamine, midazolam, and DEX) dose and in-hospital mortality in patients with COVID-19 requiring IMV in a multi-center retrospective investigation, and only DEX was associated with decreased odds of mortality, while the remaining sedatives were not associated with an increase or a decrease in odds of mortality [50].

Because this was an observational study, the timing of DEX use with regard to the start of the study (IMV) and assessment over 28 days was not standardized. Patients that started DEX later after IMV had to survive before starting DEX, which resulted in immortal time bias. Not adjusting for immortal time bias can cause substantial overestimations and underestimations of mortality outcome data [38,51,52,53]. Indeed, in this study, when looking at early vs. late DEX start times with regard to IMV, percent deaths were lower with later as opposed to earlier DEX treatment in Model 1 and Model 2; however, when DEX was treated as a time-varying covariate to adjust for immortal time bias in multivariable Cox regression, aHRs were lower with early rather than later DEX start times with regard to IMV. In this investigation, the basis of our conclusions were made treating DEX as a time-varying covariate to adjust for immortal time bias.

Addressing specific DEX associated immunomodulatory biomarkers was not planned or performed in this investigation. In clinical studies predominately evaluating DEX as a sedative in the perioperative environment or for use in critically ill patients with sepsis, DEX has been reported to decrease inflammatory markers and mediators including but not limited to the following: tumor necrosis factor alpha (TNF-α), interleukin-1 beta (IL-1β), interleukin-6 (IL-6), and C-reactive protein (CRP) [8,54]. Many of these inflammatory markers or mediators would not be possible to assess in the N3C database. However, given the overall finding of greatest lowered mortality in patients with earlier DEX use relative to IMV and the use of DEX prior to the standard utilization of dexamethasone, it would be of interest in the future to specifically evaluate markers and mediators of inflammation in this subgroup of patients. Clinical trials listed underway investigating DEX use in COVID-19 and immunomodulation with specific assessment of markers and mediators of inflammation include NCT04413864 and NCT05233605 (ClinicalTrials.Gov, accessed on 5 April 2024).

This investigation focused on mortality outcomes, and the consideration of adverse effects with DEX use was not evaluated. However, DEX can increase the risk of transient hypertension with rapid administration, bradycardia, and hypotension due to the peripheral vasoconstrictive and sympatholytic properties of the drug [55,56]. Therefore, DEX should be used with caution in specific patients [55,56].

## 5. Conclusions

In this retrospective multi-center cohort study, DEX use was associated with a reduced risk of death in patients with COVID-19 receiving IMV, particularly when initiated earlier relative to the start time of IMV and used prior to the current dexamethasone era (prior to 30 July 2020). These particular findings might suggest that DEX is improving the mortality outcomes through immunomodulatory effects. However, the immunomodulatory effects associated with DEX use were not assessed in this investigation and require further exploration. Results from a large RCT are warranted to clarify the potential mortality benefit of DEX use in COVID-19 and evaluate the physiologic changes influenced by DEX that could provide a mortality benefit.

## 6. Patents

J.L.H and M.A.W have filed a patent related to these studies (PCT/US2021/056580).

## Figures and Tables

**Table 1:** Variables at hospital admission, propensity score matched cohort (1 January 2020 to 3 November 2022).

	Model 1 ^a^			Model 2 ^b^		
	*n* = 3806			*n* = 576		
Variable	No DEX (*n* = 1903)	DEX (*n* = 1903)	SMD	No DEX (*n* = 288)	DEX (*n* = 288)	SMD
Age (SD)	63.7 (13.6)	61.6 (14.5)	0.14	64.0 (12.7)	63.1 (13.4)	0.07
Male sex (%)	1169 (61.4)	1176 (61.8)	0.01	188 (65.3)	178 (61.8)	0.07
Race (%)						
Black	325 (17.1)	337 (17.7)	0.08	<20 *	<20 *	0.11
Other	63 (3.3)	82 (4.3)		<20 *	<20 *	
Unknown	354 (18.6)	387 (20.3)		37 (12.8)	41 (14.2)	
White	1161 (61.0)	1097 (57.6)		227 (78.8)	221 (76.7)	
Ethnicity (%)						
Hispanic	329 (17.3)	344 (18.1)	0.03	43 (14.9)	31 (10.8)	0.13
Not Hispanic	1479 (77.7)	1454 (76.4)		240 (83.3)	251 (87.2)	
Unknown	95 (5.0)	105 (5.5)		<20 *	<20 *	
Active cancer (%)	179 (9.4)	163 (8.6)	0.03	24 (8.3)	22 (7.6)	0.03
Cardiovascular disease (%)						
Hypertension	1424 (74.8)	1355 (71.2)	0.08	205 (71.2)	206 (71.5)	0.01
Coronary artery disease	435 (22.9)	414 (21.8)	0.03	71 (24.7)	62 (21.5)	0.07
Congestive heart failure	465 (24.4)	457 (24.0)	0.01	80 (27.8)	71 (24.7)	0.07
Chronic respiratory disease (%)						
Asthma	225 (11.8)	217 (11.4)	0.01	32 (11.1)	30 (10.4)	0.02
COPD	590 (31.0)	573 (30.1)	0.02	97 (33.7)	92 (31.9)	0.04
Interstitial lung disease	49 (2.6)	39 (2.0)	0.04	<20 *	<20 *	0.07
Obstructive sleep apnea	370 (19.4)	355 (18.7)	0.02	53 (18.4)	64 (22.2)	0.10
Immunosuppression (%)						
HIV	<20 *	<20 *	0.03	<20 *	<20 *	0.08
Kidney disease (%)						
Chronic	495 (26.0)	451 (23.7)	0.05	64 (22.2)	52 (18.1)	0.10
End-stage	75 (3.9)	80 (4.2)	0.01	<20 *	<20 *	0.03
Liver disease (%)						
Cirrhosis	65 (3.4)	62 (3.3)	0.01	<20 *	<20 *	0.04
Hepatitis B	<20 *	<20 *	0.05	<20 *	<20 *	0.08
Hepatitis C	37 (1.9)	63 (3.3)	0.09	<20 *	<20 *	0.07
Metabolic disease						
Obesity (%)	763 (40.1)	764 (40.1)	0.00	118 (41.0)	109 (37.8)	0.06
Morbid obesity (%)	359 (18.9)	355 (18.7)	0.01	47 (16.3)	54 (18.8)	0.06
BMI (SD)	33.3 (9.6)	33.4 (9.4)	0.01	33.0 (9.6)	33.5 (9.5)	0.05
Diabetes (%)	876 (46.0)	895 (47.0)	0.02	127 (44.1)	121 (42.0)	0.04
mCCI (SD)	2.49 (2.39)	2.41 (2.39)	0.03	2.31 (2.30)	2.14 (2.15)	0.08

Abbreviations: BMI = body mass index; COPD = chronic obstructive pulmonary disease, DEX = dexmedetomidine; HIV = human immunodeficiency virus; mCCI = modified Charlson comorbidity index; SD = standard deviation; SMD = standardized mean difference. ^a^ Model 1: Health system sites included that lacked PaO_2_/FiO_2_ data. ^b^ Model 2: Only health system sites that had PaO_2_/FiO_2_ data were included. * Values less than 20 denoted as <20 as per N3C policy. Ranges used for complementary values to prevent <20 calculation.

**Table 2:** ICU variables, propensity score matched cohort (1 January 2020 to 3 November 2022).

	Model 1 ^a^			Model 2 ^b^		
	*n* = 3806			*n* = 576		
Variable	No DEX (*n* = 1903)	DEX (*n* = 1903)	SMD	No DEX (*n* = 288)	DEX (*n* = 288)	SMD
PaO_2_/FiO_2_ (SD)	--	--		85.5 (57.1)	84.8 (44.8)	0.01
mSOFA score (SD) ^c^	4.75 (2.37)	4.78 (2.23)	0.01	8.74 (1.94)	8.66 (1.75)	0.04
Sedative use (%)						
GABA receptor ligand (%)	1901 (99.9)	1878 (98.7)	0.14	288 (100.0)	278 (96.5)	0.27
Propofol	1637 (86.0)	1696 (89.1)	0.09	223 (77.4)	232 (80.6)	0.08
Midazolam	1483 (77.9)	1581 (83.1)	0.13	221 (76.7)	234 (81.2)	0.11
Lorazepam	922 (48.4)	1004 (52.8)	0.09	153 (53.1)	167 (58.0)	0.10
Ketamine	283 (14.9)	365 (19.2)	0.11	50 (17.4)	68 (23.6)	0.16
Opioid use (%)	1850 (97.2)	1858 (97.6)	0.03	280 (97.2)	281 (97.6)	0.02
Corticosteroid (any) use (%)	1559 (81.9)	1567 (82.3)	0.01	231 (80.2)	217 (75.3)	0.12
Methylprednisolone	314 (16.5)	364 (19.1)	0.07	55 (19.1)	48 (16.7)	0.06
Dexamethasone	1221 (64.2)	1249 (65.6)	0.03	173 (60.1)	171 (59.4)	0.01
Hydrocortisone	301 (15.8)	303 (15.9)	0.00	46 (16.0)	53 (18.4)	0.06
Prednisone	157 (8.3)	199 (10.5)	0.08	33 (11.5)	38 (13.2)	0.05
Remdesivir use (%)	598 (31.4)	558 (29.3)	0.05	71 (24.7)	68 (23.6)	0.02
Antibiotic (any) use (%)	1733 (91.1)	1762 (92.6)	0.06	228 (79.2)	238 (82.6)	0.09
Anticoagulant (any) use (%)	1833 (96.3)	1868 (98.2)	0.11	272 (94.4)	269 (93.4)	0.04
Heparin	1365 (71.7)	1409 (74.0)	0.05	191 (66.3)	207 (71.9)	0.12
LMWH	1285 (67.5)	1354 (71.2)	0.08	169 (58.7)	174 (60.4)	0.04
Factor Xa inhibitor	318 (16.7)	346 (18.2)	0.04	47 (16.3)	44 (15.3)	0.03
Direct thrombin inhibitor	50 (2.6)	78 (4.1)	0.08	<20 *	<20 *	0.08
Warfarin	52 (2.7)	68 (3.6)	0.05	<20 *	<20 *	0.05
Inhaled NO Use (%)	<20 *	<20 *	0.02	<20 *	<20 *	<0.01
Vasopressor Use (%)	1569 (82.4)	1685 (88.5)	0.17	254 (88.2)	263 (91.3)	0.10
Paralytic/NMB (%)	1503 (79.0)	1587 (83.4)	0.11	204 (70.8)	213 (74.0)	0.07
RRT (%)	93 (4.9)	159 (8.4)	0.14	29 (10.1)	30 (10.4)	0.01
ECMO (%)	42 (2.2)	78 (4.1)	0.11	<20 *	<20 *	0.14

Abbreviations: DEX = dexmedetomidine; ECMO = extracorporeal membrane oxygenation; GABA = gamma-aminobutyric acid; LMHW = low molecular weight heparin; mSOFA = modified Sequential Organ Failure Assessment; NMB = neuromuscular blockade; NO = nitric oxide; PaO_2_/FiO_2_ = partial pressure of arterial oxygen to the fraction of inspired oxygen; RRT = renal replacement therapy; SD = standard deviation; SMD = standardized mean difference. ^a^ Model 1: Health system sites included that lacked PaO_2_/FiO_2_ data. ^b^ Model 2: Only health system sites that had PaO_2_/FiO_2_ data were included. ^c^ mSOFA score in model 1 had the central nervous system and respiratory component removed; mSOFA score in model 2 had the central nervous system component removed. * Values of less than 20 denoted as <20 as specified by the N3C Data Enclave policy.

**Table 3:** The 28-day mortality rate from initiation of invasive mechanical ventilation with dexmedetomidine use, propensity score matched cohort (1 January 2020 to 3 November 2022).

	Model 1 ^a^		Model 2 ^b^	
	*n* = 3806		*n* = 576	
Cox Regression Model	aHR (95% CI)	*p*	aHR (95% CI)	*p*
Multivariable (DEX use) ^c^	0.81 (0.73, 0.90)	<0.001	0.95 (0.72, 1.25)	0.73
Univariable (DEX use) ^d^	0.90 (0.81, 0.99)	0.04	1.05 (0.80, 1.36)	0.74

Abbreviations: aHR = adjusted hazard ratio; DEX = dexmedetomidine; 95% CI = 95% confidence interval. ^a^ Model 1: Health system sites included that lacked PaO_2_/FiO_2_ data. ^b^ Model 2: Only health system sites that had PaO_2_/FiO_2_ data were included. ^c^ Model 1 adjusted for (i) dexmedetomidine as a time-varying covariate, (ii) age, (iii) BMI, (iv) mCCI, (v) mSOFA (central nervous system and respiratory component removed), (vi) dexamethasone use, and (vii) remdesivir use; Model 2 adjusted for (i) dexmedetomidine as a time varying covariate, (ii) age, (iii) BMI, (iv) mCCI, (v) PaO_2_/FiO_2_, (vi) mSOFA (central nervous system component removed), (vii) dexamethasone use, and (viii) remdesivir use. In both models, adjusting for site differences was performed by including a categorical variable with levels for each data partner representing >5% of our population and then all others grouped together. ^d^ Model 1 and Model 2 adjusted for dexmedetomidine use as a time-varying covariate only.

## Data Availability

Data collected with this study could be shared through the National Covid Cohort Collaborative (N3C) Data Enclave following appropriate approvals and permissions to access the data.

## Supplementary Materials

The following supporting information can be downloaded at: https://www.mdpi.com/article/10.3390/jcm13123429/s1, Supplementary Information. Table S1. Median Time Between First COVID-19 Indication and Hospitalization Following Propensity Score Matching. Table S2. Pre Propensity Score Matching Variables at Hospital Admission. Table S3. Pre Propensity Score Matching ICU Variables. Table S4. Vaccinated Prior to a COVID-19 Diagnosis (Pre Propensity Score Matching). Table S5. Interleukin-6 (IL-6) Inhibitor Use (Tocilizumab and Sarilumab) (Pre Propensity Score Matching). Table S6. Janus Kinase (JAK) Inhibitor Use (Baricitinib and Tofacitinib) (Pre Propensity Score Matching). Table S7. Vaccinated Prior to a COVID-19 Diagnosis (Post Propensity Score Matching). Table S8. Interleukin-6 (IL-6) Inhibitor Use (Tocilizumab and Sarilumab) (Post Propensity Score Matching). Table S9. Janus Kinase (JAK) Inhibitor Use (Baricitinib and Tofacitinib) (Post Propensity Score Matching. Table S10. Model 1: Multivariable Cox Regression with Dexmedetomidine as a Time-Varying Covariate (Full Study: 1 January 2020 to 3 November 2022), Propensity Score Matched Cohort. Table S11. Model 2: Multivariable Cox Regression with Dexmedetomidine as a Time-Varying Covariate (Full Study: 1 January 2020 to 3 November 2022), Propensity Score Matched Cohort. Table S12. Number and Percent Deaths in Model 1 and Model 2 in Subgroup Analysis of Early vs. Late Dexmedetomidine Start Time. Table S13. Model 1: Multivariable Cox Regression with Dexmedetomidine as a Time-Varying Covariate (Pre-Dexamethasone Era, 1 January 2020 to 30 July 2020), Propensity Score Matched Cohort. Table S14. Model 1: Multivariable Cox Regression with Dexmedetomidine as a Time-Varying Covariate (Current Dexamethasone Era, 30 July 2020 to 3 November 2022), Propensity Score Matched Cohort. Table S15. Model 2: Multivariable Cox Regression with Dexmedetomidine as a Time-Varying Covariate (Pre-Dexamethasone Era, 1 January 2020 to 30 July 2020), Propensity Score Matched Cohort. Table S16. Model 2: Multivariable Cox Regression with Dexmedetomidine as a Time-Varying Covariate (Current Dexamethasone Era, 30 July 2020 to 3 November 2022), Propensity Score Matched Cohort. Table S17. Number and Percent Death in Model 1 and Model 2 in Subgroup Analysis of Pre-Dexamethasone Era and Current Dexamethasone Era. Figure S1. Survival curve—Model 1: Multivariable Cox Regression with Dexmedetomidine as a Time-Varying Covariate (Full Study: 1 January 2020 to 3 November 2022), Propensity Score Matched Cohort. Figure S2. Survival curve—Model 2: Multivariable Cox Regression with Dexmedetomidine as a Time-Varying Covariate (Full Study: 1 January 2020 to 3 November 2022), Propensity Score Matched Cohort.
