# Supplementary material for: Association between Dexmedetomidine Use and Mortality in Patients with COVID-19 Receiving Invasive Mechanical Ventilation: A U.S. National COVID Cohort Collaborative (N3C) Study
Source: J Clin Med. 2024 Jun 12;13(12):3429. doi: 10.3390/jcm13123429 (PMC11204330; doi:10.3390/jcm13123429)

Supplementary Data

Association Between Dexmedetomidine Use and Mortality in Patients With COVID-19 Receiving Invasive Mechanical Ventilation: A US National COVID Cohort Collaborative (N3C) Study

John L. Hamilton, MD, PhD; Rachel Baccile, MPP; Thomas J. Best, PhD; Pankaja Desai, PhD; Alan Landay, PhD; Juan C. Rojas, MD, MS; Markus A. Wimmer, PhD; Robert A. Balk, MD; on behalf of the N3C Consortium

Table S1. Median Time Between First COVID-19 Indication and Hospitalization Following Propensity Score Matching
Table S2. Pre Propensity Score Matching Variables at Hospital Admission
Table S3. Pre Propensity Score Matching ICU Variables
Table S4. Vaccinated Prior to a COVID-19 Diagnosis (Pre Propensity Score Matching)
Table S5. Interleukin-6 (IL-6) Inhibitor Use (Tocilizumab and Sarilumab) (Pre Propensity Score Matching)
Table S6. Janus Kinase (JAK) Inhibitor Use (Baricitinib and Tofacitinib) (Pre Propensity Score Matching)
Table S7. Vaccinated Prior to a COVID-19 Diagnosis (Post Propensity Score Matching)
Table S8. Interleukin-6 (IL-6) Inhibitor Use (Tocilizumab and Sarilumab) (Post Propensity Score Matching)
Table S9. Janus Kinase (JAK) Inhibitor Use (Baricitinib and Tofacitinib) (Post Propensity Score Matching
Table S10. Model 1: Multivariable Cox Regression With Dexmedetomidine as a Time-Varying Covariate (Full Study: January 1, 2020 to November 3, 2022), Propensity Score Matched Cohort
Table S11. Model 2: Multivariable Cox Regression With Dexmedetomidine as a Time-Varying Covariate (Full Study: January 1, 2020 to November 3, 2022), Propensity Score Matched Cohort
Table S12. Number and Percent Deaths in Model 1 and Model 2 in Subgroup Analysis of Early vs. Late Dexmedetomidine Start Time
Table S13. Model 1: Multivariable Cox Regression With Dexmedetomidine as a Time-Varying Covariate (Pre-Dexamethasone Era, January 1, 2020 to July 30, 2020), Propensity Score Matched Cohort
Table S14. Model 1: Multivariable Cox Regression With Dexmedetomidine as a Time-Varying Covariate (Current Dexamethasone Era, July 30, 2020 to November 3, 2022), Propensity Score Matched Cohort
Table S15. Model 2: Multivariable Cox Regression With Dexmedetomidine as a Time-Varying Covariate (Pre-Dexamethasone Era, January 1, 2020 to July 30, 2020), Propensity Score Matched Cohort
Table S16. Model 2: Multivariable Cox Regression With Dexmedetomidine as a Time-Varying Covariate (Current Dexamethasone Era, July 30, 2020 to November 3, 2022), Propensity Score Matched Cohort
Table S17. Number and Percent Death in Model 1 and Model 2 in Subgroup Analysis of Pre-Dexamethasone Era and Current Dexamethasone Era
Figure S1. Survival curve - Model 1: Multivariable Cox Regression With Dexmedetomidine as a Time-Varying Covariate (Full Study: January 1, 2020 to November 3, 2022), Propensity Score Matched Cohort
Figure S2. Survival curve - Model 2: Multivariable Cox Regression With Dexmedetomidine as a Time-Varying Covariate (Full Study: January 1, 2020 to November 3, 2022), Propensity Score Matched Cohort

Table S1. Median Time Between First COVID-19 Indication and Hospitalization Following Propensity Score Matching

	**DEX**	**No DEX**	***P***
Model 1 days from positive test to hospitalization (SD)	1.11 (2.8)	1.16 (2.7)	0.58
Model 2 days from positive test to hospitalization (SD)	1.32 (2.9)	0.84 (2.0)	0.02

Abbreviations: SD = standard deviation

Table S2. Pre Propensity Score Matching Variables at Hospital Admission

	Model 1[a]			Model 2[b]		
	N = 6,109			*N* = 955		
**Variable**	**No DEX (*n* = 3,107)**	**DEX (*n* = 3,002)**	**SMD**	**No DEX (*n* = 340)**	**DEX (*n* = 615)**	**SMD**
Age (SD)	63.4 (14.1)	59.1 (15.0)	0.30	63.2 (14.0)	58.6 (14.6)	0.32
Male Sex (%)	1999 (64.3)	1893 (63.1)	0.03	221 (65.0)	383 (62.3)	0.06
Race (%)						
Black	450 (14.5)	476 (15.9)	0.08	< 20*	22 (3.6)	0.18
Other	182 (5.9)	129 (4.3)		< 20*	50 (8.1)	
Unknown	717 (23.1)	727 (24.2)		52 (15.3)	90 (14.6)	
White	1758 (56.6)	1670 (55.6)		261 (76.8)	453 (73.7)	
Ethnicity (%)						
Hispanic	552 (17.8)	679 (22.6)	0.13	45-65 (13-19)*	79 (12.8)	0.13
Not Hispanic	2325 (74.8)	2150 (71.6)		277 (81.5)	514 (83.6)	
Unknown	230 (7.4)	173 (5.8)		< 20*	22 (3.6)	
Active Cancer (%)	248 (8.6)	207 (6.9)	0.04	28 (8.2)	31 (5.0)	0.13
Cardiovascular Disease (%)						
Hypertension	2247 (72.3)	1971 (65.7)	0.14	228 (67.1)	351 (57.1)	0.21
Coronary Artery Disease	702 (22.6)	543 (18.1)	0.11	78 (22.9)	99 (16.1)	0.17
Congestive Heart Failure	678 (21.8)	661 (22.0)	< 0.01	83 (24.4)	113 (18.4)	0.15
Chronic respiratory disease (%)						
Asthma	350 (11.3)	336 (11.2)	< 0.01	34 (10.0)	60 (9.8)	0.01
COPD	803 (27.9)	798 (28.4)	0.01	103 (30.3)	162 (26.3)	0.09
Interstitial Lung Disease	72 (2.4)	68 (2.4)	0.01	< 20*	24 (3.9)	0.01
Apnea	489 (15.7)	510 (17.0)	0.03	57 (16.8)	115 (18.7)	0.05
Immunosuppression (%)						
HIV	29 (0.9)	33 (1.1)	0.02	< 20*	< 20	0.03
Kidney Disease (%)						
Chronic	811 (26.1)	640 (21.3)	0.11	71 (20.9)	96 (15.6)	0.14
End-Stage	144 (4.6)	124 (4.1)	0.02	< 20*	< 20*	0.03
Liver Disease (%)						
Cirrhosis	85 (2.7)	95 (3.2)	0.03	< 20*	< 20*	0.03
Hepatitis B	< 20*	< 20*	0.01	< 20*	< 20*	0.08
Hepatitis C	48 (1.5)	93 (3.1)	0.10	< 20*	< 20*	0.08
Metabolic Disease						
Obesity (%)	1198 (38.6)	1197 (39.9)	0.03	136 (40.0)	237 (38.5)	0.03
Morbid Obesity (%)	495 (15.9)	544 (18.1)	0.06	55 (16.2)	122 (19.8)	0.10
BMI (SD)	32.3 (9.1)	33.2 (9.3)	0.10	32.7 (9.4)	33.7 (9.2)	0.10
Diabetes (%)	1374 (44.2)	1301 (43.3)	0.02	137 (40.3)	226 (36.7)	0.07
mCCI (SD)	2.5 (2.4)	2.3 (2.3)	0.08	2.2 (2.3)	1.8 (2.0)	0.19

Abbreviations: BMI = body mass index; COPD = chronic obstructive pulmonary disease, DEX = dexmedetomidine; HIV = human immunodeficiency virus; mCCI = modified Charlson Comorbidity Index; SD = standard deviation; SMD = standardized mean difference

[a] Model 1: Health system sites included that lacked PaO2/FiO2 data

[b] Model 2: Only health system sites that had PaO2/FiO2 data were included

* Values less than 20 denoted as < 20 per N3C policy. Ranges used for complementary values to prevent < 20 calculation.

Table S3. Pre Propensity Score Matching ICU Variables

	**Model 1[a]**			**Model 2[b]**		
	***N* = 6,109**			***N* = 955**		
**Variable**	**No DEX (*n* = 3,107)**	**DEX (*n* = 3,002)**	**SMD**	**No DEX (*n* = 340)**	**DEX (*n* = 615)**	**SMD**
PaO2/FiO2 (SD)	--	--		89.1 (69.6)	83.1 (41.3)	0.10
mSOFA score (SD)[c]	4.8 (2.3)	4.7 (2.2)	0.05	8.7 (2.0)	8.6 (1.7)	0.10
Sedative Use (%)						
GABA receptor ligand (%)	3091 (99.5)	2950 (98.3)	0.12	331 (97.4)	592 (96.3)	0.06
Propofol	2576 (82.9)	2670 (88.9)	0.17	256 (75.3)	487 (79.2)	0.09
Midazolam	2432 (78.3)	2558 (85.2)	0.18	259 (76.2)	515 (83.7)	0.19
Lorazepam	1656 (53.3)	1582 (52.7)	0.01	185 (54.4)	364 (59.2)	0.10
Ketamine	509 (16.4)	742 (24.7)	0.21	61 (17.9)	239 (38.9)	0.48
Opioid use (%)	2876 (92.6)	2917 (97.2)	0.21	322 (94.7)	591 (96.1)	0.07
Corticosteroid (any) use (%)	2464 (79.3)	2497 (83.2)	0.10	268 (78.8)	491 (79.8)	0.03
Methylprednisolone	714 (23.0)	595 (19.8)	0.08	68 (20.0)	97 (15.8)	0.11
Dexamethasone	1683 (54.2)	1921 (64.0)	0.20	198 (58.2)	408 (66.3)	0.17
Hydrocortisone	632 (20.3)	706 (23.5)	0.08	75 (22.7)	162 (26.6)	0.09
Prednisone	234 (7.5)	288 (9.6)	0.07	36 (10.6)	93 (15.1)	0.14
Remdesivir use (%)	696 (22.4)	870 (29.0)	0.15	84 (24.7)	193 (31.4)	0.15
Antibiotic (any) use (%)	2451 (78.9)	2805 (93.4)	0.43	259 (76.2)	542 (88.1)	0.32
Anticoagulant (any) use (%)	2987 (96.1)	2928 (97.5)	0.08	309 (90.9)	584 (95.0)	0.16
Heparin	2343 (75.4)	2230 (74.3)	0.03	215 (63.2)	426 (69.3)	0.13
LMWH	2115 (68.1)	2179 (72.6)	0.10	200 (58.8)	373 (60.7)	0.04
Factor Xa inhibitor	487 (15.7)	536 (17.9)	0.06	49 (14.4)	105 (17.1)	0.07
Direct thrombin inhibitor	54 (1.7)	204 (6.8)	0.25	< 20*	68 (11.1)	0.37
Warfarin	71 (2.3)	107 (3.6)	0.08	< 20*	36 (5.9)	0.08
Inhaled NO Use (%)	< 20*	< 20*	0.01	< 20*	< 20*	0.03
Vasopressor Use (%)	2677 (86.2)	2655 (88.4)	0.07	286 (84.1)	553 (89.9)	0.17
Paralytic / NMB (%)	2644 (85.1)	2672 (89.0)	0.12	236 (69.4)	508 (82.6)	0.31
RRT (%)	282 (9.1)	295 (9.8)	0.03	29 (8.5)	98 (15.9)	0.23
ECMO (%)	114 (3.7)	203 (6.8)	0.14	< 20*	50 (8.1)	0.38

Abbreviations: DEX = dexmedetomidine; ECMO = extracorporeal membrane oxygenation; GABA = gamma-aminobutyric acid; LMHW = low molecular weight heparin; mSOFA= modified Sequential Organ Failure Assessment; NMB = neuromuscular blockade; NO = nitric oxide; PaO2/FiO2 = partial pressure of arterial oxygen to the fraction of inspired oxygen; RRT = renal replacement therapy; SD = standard deviation; SMD = standardized mean difference

[a] Model 1: Health system sites included that lacked PaO2/FiO2 data

[b] Model 2: Only health system sites that had PaO2/FiO2 data were included

[c] mSOFA score in Model 1 has central nervous system and respiratory component removed; mSOFA score in Model 2 has central nervous system component removed

* Values of less than 20 denoted as < 20 as specified by N3C Data Enclave policy

**Table S4.** Vaccinated Prior to a COVID-19 Diagnosis (Pre-Propensity Score Matching)						
	**Model 1[a]**			**Model 2[b]**		
	***N* = 6,109**			***N* = 955**		
**Variable**	**No DEX (*n* = 3,107)**	**DEX (*n* = 3,002)**	**SMD**	**No DEX (*n* = 340)**	**DEX (*n* = 615)**	**SMD**
Vaccinated (%)	78 (2.5)	78 (2.6)	< 0.01	14 (4.1)	12 (2.0)	0.13

Abbreviations: DEX = dexmedetomidine; SMD = standardized mean difference
[a] Model 1: Health system sites included that lacked PaO2/FiO2 data
[b] Model 2: Only health system sites that had PaO2/FiO2 data were included

**Table S5.** Interleukin-6 (IL-6) Inhibitor Use (Tocilizumab and Sarilumab) (Pre-Propensity Score Matching)						
	**Model 1[a]**			**Model 2[b]**		
	***N* = 6,109**			***N* = 955**		
**Variable**	**No DEX (*n* = 3,107)**	**DEX (*n* = 3,002)**	**SMD**	**No DEX (*n* = 340)**	**DEX (*n* = 615)**	**SMD**
IL-6 inhibitor (%)	130 (4.2)	167 (5.6)	0.06	< 20*	39 (6.3)	0.10

Abbreviations: DEX = dexmedetomidine; SMD = standardized mean difference
[a] Model 1: Health system sites included that lacked PaO2/FiO2 data
[b] Model 2: Only health system sites that had PaO2/FiO2 data were included
* Values of less than 20 denoted as < 20 as specified by N3C Data Enclave policy

**Table S6.** Janus Kinase (JAK) Inhibitor Use (Baricitinib and Tofacitinib) (Pre-Propensity Score Matching)						
	**Model 1[a]**			**Model 2[b]**		
	***N* = 6,109**			***N* = 955**		
**Variable**	**No DEX (*n* = 3,107)**	**DEX (*n* = 3,002)**	**SMD**	**No DEX (*n* = 340)**	**DEX (*n* = 615)**	**SMD**
JAK inhibitor (%)	< 20*	< 20*	< 0.001	< 20*	< 20*	< 0.001

Abbreviations: DEX = dexmedetomidine; SMD = standardized mean difference
[a] Model 1: Health system sites included that lacked PaO2/FiO2 data
[b] Model 2: Only health system sites that had PaO2/FiO2 data were included
* Values of less than 20 denoted as < 20 as specified by N3C Data Enclave policy

**Table S7.** Vaccinated Prior to a COVID-19 Diagnosis (Post-Propensity Score Matching)						
	**Model 1[a]**			**Model 2[b]**		
	***N* = 3,806**			***N* = 576**		
**Variable**	**No DEX (*n* = 1,903)**	**DEX (*n* = 1,903)**	**SMD**	**No DEX (*n* = 288)**	**DEX (*n* = 288)**	**SMD**
Vaccinated (%)	73 (3.8)	65 (3.4)	0.02	< 20*	< 20*	0.16

Abbreviations: DEX = dexmedetomidine; SMD = standardized mean difference
[a] Model 1: Health system sites included that lacked PaO2/FiO2 data
[b] Model 2: Only health system sites that had PaO2/FiO2 data were included
* Values of less than 20 denoted as < 20 as specified by N3C Data Enclave policy

**Table S8.** Interleukin-6 (IL-6) Inhibitor Use (Tocilizumab and Sarilumab) (Post-Propensity Score Matching)						
	**Model 1[a]**			**Model 2[b]**		
	***N* = 3,806**			***N* = 576**		
**Variable**	**No DEX (*n* = 1,903)**	**DEX (*n* = 1,903)**	**SMD**	**No DEX (*n* = 288)**	**DEX (*n* = 288)**	**SMD**
IL-6 inhibitor (%)	106 (5.6)	104 (5.5)	< 0.01	< 20*	< 20*	0.19

Abbreviations: DEX = dexmedetomidine; SMD = standardized mean difference
[a] Model 1: Health system sites included that lacked PaO2/FiO2 data
[b] Model 2: Only health system sites that had PaO2/FiO2 data were included
* Values of less than 20 denoted as < 20 as specified by N3C Data Enclave policy

**Table S9.** Janus Kinase (JAK) Inhibitor Use (Baricitinib and Tofacitinib) (Post-Propensity Score Matching)						
	**Model 1[a]**			**Model 2[b]**		
	***N* = 3,806**			***N* = 576**		
**Variable**	**No DEX (*n* = 1,903)**	**DEX (*n* = 1,903)**	**SMD**	**No DEX (*n* = 288)**	**DEX (*n* = 288)**	**SMD**
JAK inhibitor (%)	< 20*	< 20*	< 0.001	< 20*	< 20*	< 0.001

Abbreviations: DEX = dexmedetomidine; SMD = standardized mean difference
[a] Model 1: Health system sites included that lacked PaO2/FiO2 data
[b] Model 2: Only health system sites that had PaO2/FiO2 data were included
* Values of less than 20 denoted as < 20 as specified by N3C Data Enclave policy

Table S10: Model 1: Multivariable Cox Regression With Dexmedetomidine as a Time-Varying Covariate (Full Study: January 1, 2020 to November 3, 2022), Propensity Score Matched Cohort

	**DEX use (any time) (*n* = 3,806)**		**DEX use (<=3.5 days) (*n* = 2,857)**		**DEX use (>3.5 days) (*n* = 2,852)**	
	**aHR (95%CI)**	***P***	**aHR (95%CI)**	***P***	**aHR (95%CI)**	***P***
DEX exposure	0.81 (0.73, 0.9)	< 0.01	0.67 (0.6, 0.76)	< 0.01	0.71 (0.61, 0.83)	< 0.01
mCCI	1.03 (1.01, 1.05)	< 0.01	1.03 (1, 1.05)	0.03	1.04 (1.01, 1.06)	< 0.01
BMI	1 (1, 1.01)	0.10	1.01 (1, 1.02)	< 0.01	1 (1, 1.01)	0.38
Age at COVID hospitalization	1.04 (1.04, 1.04)	< 0.01	1.04 (1.03, 1.04)	< 0.01	1.04 (1.03, 1.04)	< 0.01
mSOFA[a]	1.15 (1.12, 1.18)	< 0.01	1.15 (1.12, 1.19)	< 0.01	1.15 (1.12, 1.19)	< 0.01
Dexamethasone exposure	1.08 (0.97, 1.21)	0.17	1.05 (0.93, 1.18)	0.47	1.01 (0.88, 1.14)	0.94
Remdesivir exposure	0.9 (0.81, 1.01)	0.08	0.91 (0.81, 1.03)	0.14	0.88 (0.77, 0.99)	0.04
Data partner 1	1.54 (1.24, 1.92)	< 0.01	1.58 (1.26, 2)	< 0.01	1.41 (1.04, 1.92)	0.03
Data partner 2	0.68 (0.59, 0.78)	< 0.01	0.72 (0.61, 0.85)	< 0.01	0.66 (0.56, 0.77)	< 0.01
Data partner 3	0.68 (0.56, 0.83)	< 0.01	0.65 (0.52, 0.82)	< 0.01	0.74 (0.59, 0.93)	0.01
Data partner 4	1.09 (0.92, 1.29)	0.34	1.22 (1.01, 1.46)	0.04	1.02 (0.83, 1.25)	0.85
Data partner 5	0.66 (0.56, 0.79)	< 0.01	0.83 (0.69, 1)	0.05	0.68 (0.56, 0.82)	< 0.01
Data partner other	0.41 (0.34, 0.5)	< 0.01	0.43 (0.36, 0.53)	< 0.01	0.38 (0.31, 0.47)	< 0.01

Abbreviations: BMI = body mass index; DEX = dexmedetomidine; mCCI = modified Charlson Comorbidty Index; mSOFA = modified Sequential Organ Failure Assessment

[a] mSOFA score modified with central nervous system and respiratory component removed

Table S11: Model 2: Multivariable Cox Regression with Dexmedetomidine as a Time-Varying Covariate (Full Study: January 1, 2020 to November 3, 2022), Propensity Score Matched Cohort

	**DEX use (any time) (*n* = 576)**		**DEX use (<=4 days) (*n* = 448)**		**DEX use (>4 days) (*n* = 416)**	
	**aHR (95%CI)**	***P***	**aHR (95%CI)**	***P***	**aHR (95%CI)**	***P***
DEX exposure	0.95 (0.72, 1.25)	0.73	0.72 (0.53, 0.98)	0.04	0.97 (0.65, 1.45)	0.87
mCCI	1.09 (1.02, 1.16)	0.01	1.08 (1.01, 1.16)	0.03	1.11 (1.04, 1.19)	< 0.01
BMI	0.99 (0.98, 1.01)	0.21	1 (0.98, 1.01)	0.83	1 (0.98, 1.01)	0.58
Age at COVID hospitalization	1.03 (1.02, 1.05)	< 0.01	1.04 (1.02, 1.05)	< 0.01	1.04 (1.02, 1.05)	< 0.01
PaO2/FiO2	0.99 (0.99, 1)	0.01	0.99 (0.99, 1)	0.01	0.99 (0.99, 1)	0.04
mSOFA[a]	1.16 (1.08, 1.24)	< 0.01	1.17 (1.09, 1.26)	< 0.01	1.16 (1.07, 1.26)	< 0.01
Dexamethasone exposure	1.09 (0.84, 1.41)	0.50	1.05 (0.79, 1.38)	0.75	1.11 (0.81, 1.53)	0.50
Remdesivir exposure	0.88 (0.64, 1.21)	0.43	0.77 (0.55, 1.09)	0.14	0.85 (0.59, 1.24)	0.40
Data partner 3	0.71 (0.53, 0.96)	0.03	0.57 (0.4, 0.81)	< 0.01	0.79 (0.57, 1.11)	0.17
data partner 6	0.47 (0.33, 0.65)	< 0.01	0.4 (0.28, 0.59)	< 0.01	0.4 (0.27, 0.61)	< 0.01
data partner other	0.18 (0.02, 1.49)	0.11	0.55 (0.07, 4.3)	0.57	0.17 (0.02, 1.51)	0.11

Abbreviations: BMI = body mass index; DEX = dexmedetomidine; mCCI = modified Charlson Comorbidty Index; mSOFA = modified Sequential Organ Failure Assessment; PaO2/FiO2 = partial pressure of arterial oxygen to the fraction of inspired oxygen

[a] mSOFA score modified with central nervous system component removed

Table S12: Number and Percent Deaths in Model 1 and Model 2 in Subgroup Analysis of Early vs Late Dexmedetomidine Start time

	**DEX group**			**No DEX group**			
**Model and subgroup**	**Patients (*n*)**	**Deaths (*n*)**	**Deaths (%)**	**Patients (*n*)**	**Deaths (*n*)**	**Deaths (%)**	***P***
Model 1; Early [a]	954	541	56.71	1903	1275	67.00	<0.01
Model 1; Late [b]	949	361	38.04	1903	1275	67.00	<0.01
Model 2: Early [c]	160	87	54.38	288	176	61.11	0.01
Model 2: Late [d]	128	56	43.75	288	176	61.11	0.20

[a] Dexmedetomidine start time < 3.5 days from the initiation of invasive mechanical ventilation
[b] Dexmedetomidine start time > 3.5 days from the initiation of invasive mechanical ventilation
[c] Dexmedetomidine start time < 4.0 days from the initiation of invasive mechanical ventilation
[d] Dexmedetomidine start time > 4.0 days from the initiation of invasive mechanical ventilation

**Table S13:** Model 1: Multivariable Cox Regression With Dexmedetomidine as a Time-Varying Covariate (Pre-Dexamethasone Era, January 1, 2020 to July 30, 2020), Propensity Score Matched Cohort		
	**DEX use (any time) (*n* = 909)**	
	**aHR (95%CI)**	***P***
DEX exposure	0.54 (0.42, 0.69)	< 0.01
mCCI	1.08 (1.03, 1.14)	< 0.01
BMI	1 (0.99, 1.02)	0.76
Age at COVID hospitalization	1.05 (1.04, 1.06)	< 0.01
mSOFA[a]	1.21 (1.14, 1.28)	< 0.01
Dexamethasone exposure	0.88 (0.65, 1.19)	0.41
Remdesivir exposure	0.97 (0.63, 1.48)	0.89
Data partner 1	1.72 (0.77, 3.85)	0.19
Data partner 2	0.93 (0.62, 1.4)	0.73
Data partner 3	1.18 (0.7, 1.99)	0.54
Data partner 4	1.15 (0.72, 1.82)	0.57
Data partner 5	1 (0.68, 1.47)	1.00
Data partner other	0.65 (0.42, 1.01)	0.06

Abbreviations: BMI = body mass index; DEX = dexmedetomidine; mCCI = modified Charlson Comorbidty Index; mSOFA = modified Sequential Organ Failure Assessment
[a] mSOFA score modified with central nervous system and respiratory component removed

**Table S14:** Model 1: Multivariable Cox Regression With Dexmedetomidine as a Time-Varying Covariate (Current Dexamethasone Era, July 30, 2020 to November 3, 2022), Propensity Score Matched Cohort		
	**DEX use (any time) (*n* = 2,897)**	
	**aHR (95%CI)**	***P***
DEX exposure	0.89 (0.79, 1)	0.06
mCCI	1.01 (0.99, 1.04)	0.25
BMI	1.01 (1, 1.01)	0.06
Age at COVID hospitalization	1.04 (1.03, 1.04)	< 0.01
mSOFA[a]	1.13 (1.09, 1.16)	< 0.01
Dexamethasone exposure	0.88 (0.76, 1.01)	0.08
Remdesivir exposure	0.86 (0.76, 0.96)	0.01
Data partner 1	1.38 (1.09, 1.73)	0.01
Data partner 2	0.64 (0.55, 0.75)	< 0.01
Data partner 3	0.58 (0.47, 0.72)	< 0.01
Data partner 4	1.11 (0.93, 1.34)	0.24
Data partner 5	0.61 (0.49, 0.76)	< 0.01
Data partner other	0.37 (0.3, 0.46)	< 0.01

Abbreviations: BMI = body mass index; DEX = dexmedetomidine; mCCI = modified Charlson Comorbidty Index; mSOFA = modified Sequential Organ Failure Assessment
[a] mSOFA score modified with central nervous system and respiratory component removed

**Table S15:** Model 2: Multivariable Cox regression with Dexmedetomidine as a Time-Varying Covariate (Pre-Dexamethasone Era, January 1, 2020 to July 30, 2020), Propensity Score Matched Cohort		
	**DEX use (any time) (*n* = 73)**	
	**aHR (95%CI)**	***P***
DEX exposure	0.22 (0.06, 0.78)	0.02
mCCI	1.19 (0.89, 1.59)	0.25
BMI	0.97 (0.89, 1.06)	0.53
Age at COVID hospitalization	1.08 (1.03, 1.13)	< 0.01
PaO2/FiO2	1 (0.99, 1.01)	0.86
mSOFA[a]	1.2 (0.89, 1.62)	0.24
Dexamethasone exposure	1.4 (0.44, 4.48)	0.57
Remdesivir exposure	1.84 (0.74, 4.58)	0.19
Data partner 3	0.49 (0.21, 1.16)	0.11
data partner 6	0.43 (0.16, 1.17)	0.10
data partner other	0 (0, 0)	< 0.01

Abbreviations: BMI = body mass index; DEX = dexmedetomidine; mCCI = modified Charlson Comorbidty Index; mSOFA = modified Sequential Organ Failure Assessment; PaO2/FiO2= partial pressure of arterial oxygen to the fraction of inspired oxygen
[a] mSOFA score modified with central nervous system component removed

**Table S16:** Model 2: Multivariable Cox Regression With Dexmedetomidine as a Time-Varying Covariate (Current Dexamethasone Era, July 30, 2020 to November 3, 2022), Propensity Score Matched Cohort		
	**DEX use (any time) (*n* = 503)**	
	**aHR (95%CI)**	***P***
DEX exposure	1.03 (0.77, 1.38)	0.83
mCCI	1.07 (1, 1.15)	0.06
BMI	0.99 (0.98, 1.01)	0.30
Age at COVID hospitalization	1.03 (1.02, 1.05)	< 0.01
PaO2/FiO2	0.99 (0.99, 1)	0.02
mSOFA[a]	1.16 (1.08, 1.24)	< 0.01
Dexamethasone exposure	0.99 (0.74, 1.31)	0.94
Remdesivir exposure	0.87 (0.62, 1.22)	0.42
Data partner 3	0.73 (0.52, 1.02)	0.07
data partner 6	0.5 (0.35, 0.71)	< 0.01
data partner other	0.28 (0.03, 2.6)	0.26

Abbreviations: BMI = body mass index; DEX = dexmedetomidine; mCCI = modified Charlson Comorbidty Index; mSOFA = modified Sequential Organ Failure Assessment; PaO2/FiO2= partial pressure of arterial oxygen to the fraction of inspired oxygen
[a] mSOFA score modified with central nervous system component removed

Table S17: Number and Percent Death in Model 1 and Model 2 in Subgroup Analysis of Pre-Dexamethasone Era and Current Dexamethasone Era

	DEX group			No DEX group			
**Model and subgroup**	**Patients (*n*)**	**Deaths (*n*)**	**Deaths (%)**	**Patients (*n*)**	**Deaths (*n*)**	**Deaths (%)**	***P***
Model 1; Pre-Dexamethasone Era [a]	483	175	36.23	426	267	62.68	<0.01
Model 1; Current Dexamethasone Era [b]	1420	727	51.20	1477	1008	68.25	<0.01
Model 2: Pre-Dexamethasone Era [a]	31	< 20	masked	42	25	59.52	0.13
Model 2: Current Dexamethasone Era [b]	257	131	50.97	246	151	61.38	0.02

[a] Pre-Dexamethasone Era were patients assessed between 1/1/20 – 7/30/2020
[b] Current Dexamethasone Era were patients assessed between 7/30/2020 – 10/3/2022

Figure S1: Survival curve - Model 1: Multivariable Cox Regression With Dexmedetomidine as a Time-Varying Covariate (Full Study: January 1, 2020 to November 3, 2022), Propensity Score Matched Cohort

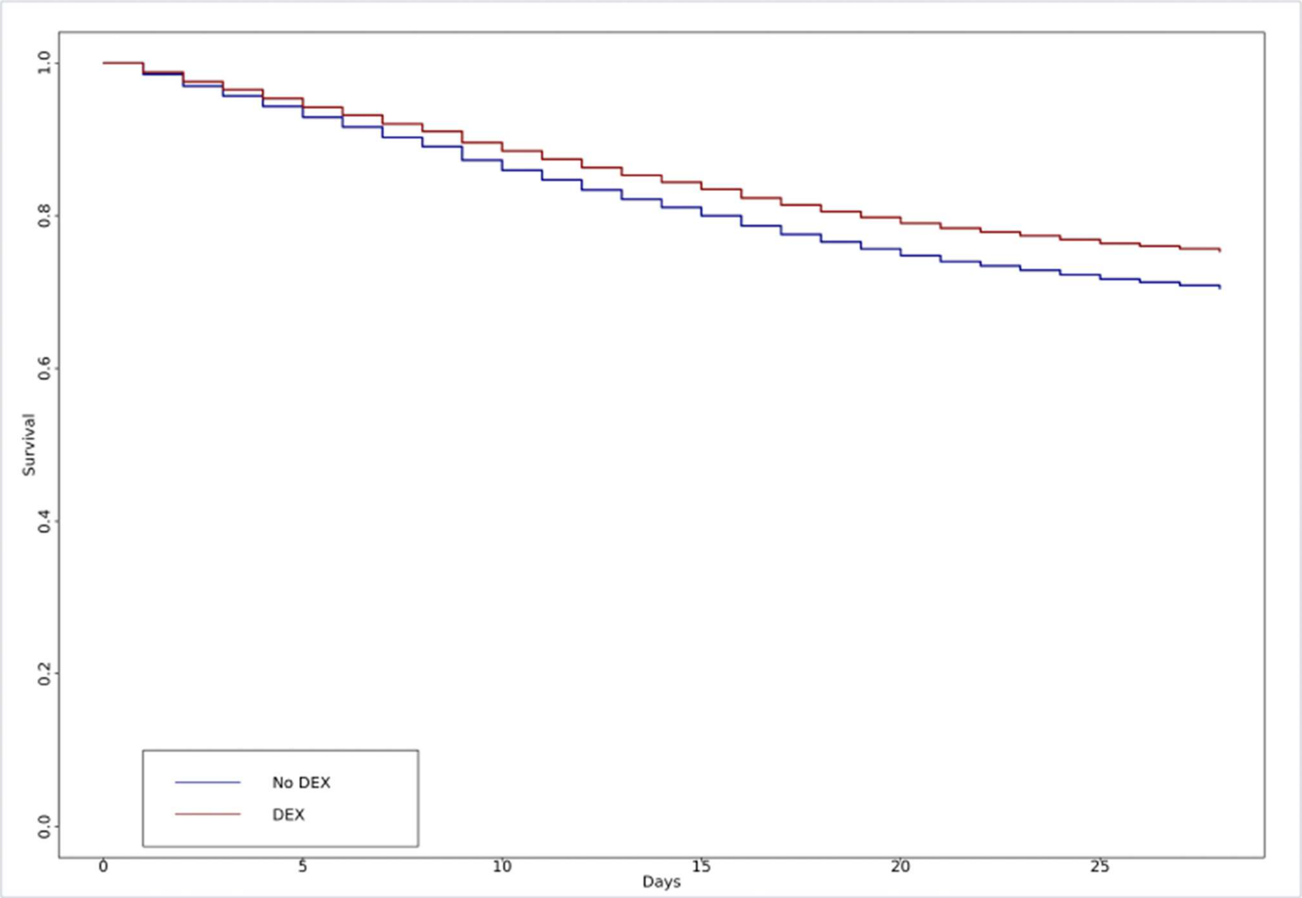

Figure S2: Survival curve - Model 2: Multivariable Cox Regression With Dexmedetomidine as a Time-Varying Covariate (Full Study: January 1, 2020 to November 3, 2022), Propensity Score Matched Cohort

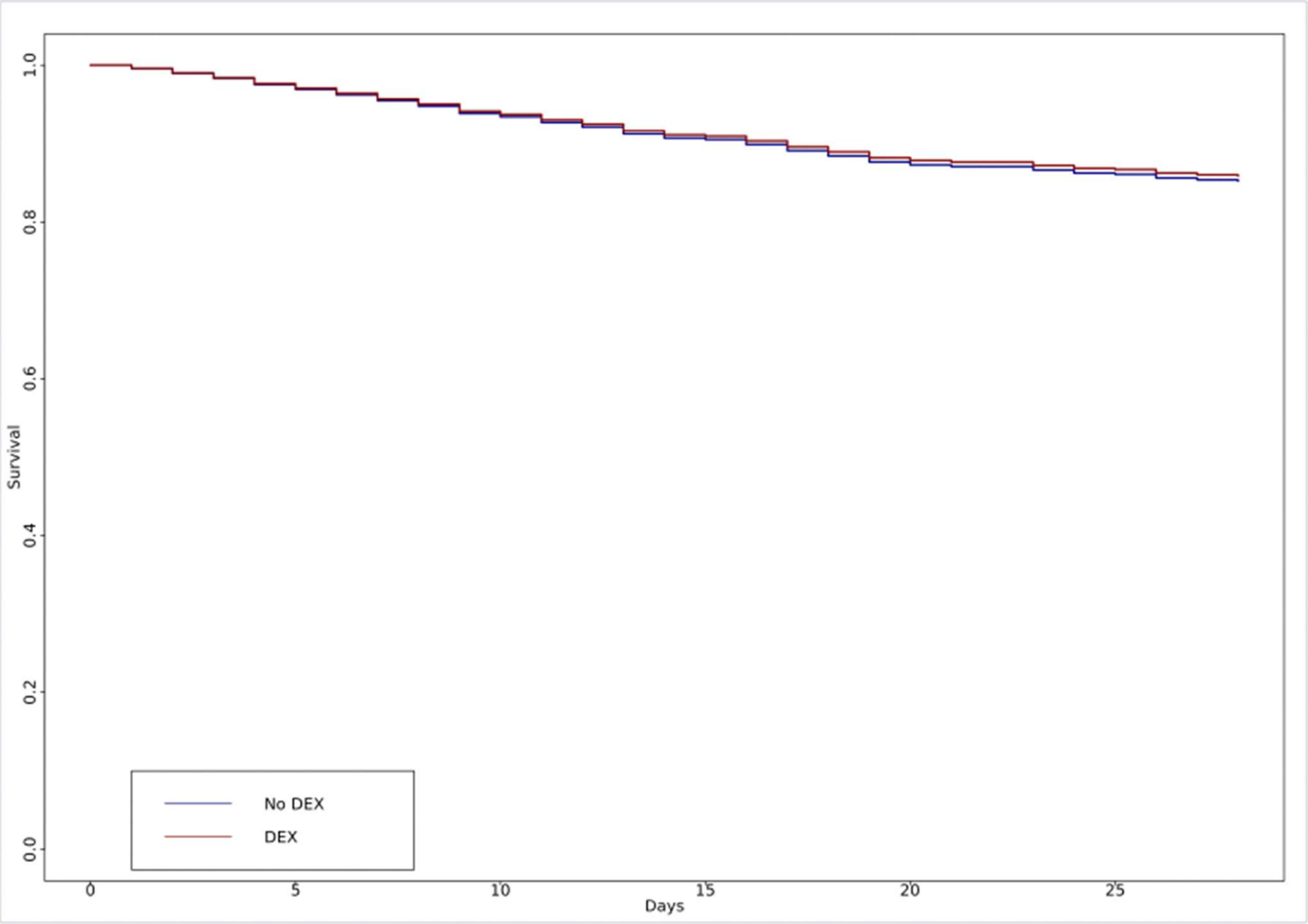

Supplement: Supplementary file 1 [file jcm-13-03429-s001.zip › jcm-2990685-supplementary.pdf]
